# Laser-induced thermoelastic effects can evoke tactile sensations

**DOI:** 10.1038/srep11016

**Published:** 2015-06-05

**Authors:** Jae-Hoon Jun, Jong-Rak Park, Sung-Phil Kim, Young Min Bae, Jang-Yeon Park, Hyung-Sik Kim, Seungmoon Choi, Sung Jun Jung, Seung Hwa Park, Dong-Il Yeom, Gu-In Jung, Ji-Sun Kim, Soon-Cheol Chung

**Affiliations:** 1 Department of Biomedical Engineering, BK21+ Research Institute of Biomedical Engineering, College of Biomedical & Health Science, Konkuk University, Chungju, Chungbuk 380-701, South Korea; 2 Department of Photonic Engineering, Chosun University, Gwangju 501-759, South Korea; 3 Department of Human and Systems Engineering, Ulsan National Institute of Science and Technology, Ulsan 689-798, South Korea; 4 Department of Physiology, KU Open Innovation Center, Research Institute of Medical Science, Konkuk University School of Medicine, Chungju, Chungbuk 380-701, South Korea; 5 Center for Neuroscience Imaging Research (CNIR), Institute for Basic Science (IBS), Suwon, Gyeonggi 440-746, South Korea; 6 Department of Biomedical Engineering, Sungkyunkwan University, Suwon, Gyeonggi 440-746, South Korea; 7 Department of Computer Science and Engineering, Pohang University of Science and Technology, Pohang, Gyeongbuk 790-784, South Korea; 8 Department of Physiology, Medical School, Hanyang University, Seoul 133-791, South Korea; 9 Department of Anatomy, Konkuk University School of Medicine, Chungju, Chungbuk 380-701, South Korea; 10 Department of Physics and Energy Systems Research, Ajou University, Suwon, Gyeonggi 443-749, South Korea

## Abstract

Humans process a plethora of sensory information that is provided by various entities in the surrounding environment. Among the five major senses, technology for touch, haptics, is relatively young and has relatively limited applications largely due to its need for physical contact. In this article, we suggest a new way for *non-contact* haptic stimulation that uses *laser*, which has potential advantages such as mid-air stimulation, high spatial precision, and long working distance. We demonstrate such tactile stimulation can be enabled by laser-induced thermoelastic effects by means of physical and perceptual studies, as well as simulations. In the physical study, the mechanical effect of laser on a human skin sample is detected using low-power radiation in accordance with safety guidelines. Limited increases (< ~2.5 °C) in temperature at the surface of the skin, examined by both thermal camera and the Monte Carlo simulation, indicate that laser does not evoke heat-induced nociceptive sensation. In the human EEG study, brain responses to both mechanical and laser stimulation are consistent, along with subjective reports of the non-nociceptive sensation of laser stimuli.

Our life is contingent upon processing a multitude of sensory information coming from our environment. Among the five major human senses, technologies for creating synthetic visual and auditory stimuli have progressed immensely with critical impact to our society. These are followed by the technology for touch, called haptics, which is relatively young and has begun to be adopted in some special areas such as robotic surgery, virtual reality-based training, collision warning in vehicles, touchscreen feedback, and games. A particular requirement hindering further spread of haptic technology is that tactile stimuli must be delivered through direct contact with the skin. This need for contact limits the diversity of haptic applications and has led to the advent of so-called non-contact haptics. In non-contact haptics, stimulus energy is transmitted without a solid medium, thereby enabling contactless tactile stimulation. To this end, recent research tested the feasibility of using an array of ultrasonic transducers^1^ or tight vortices of air^2^. Although these approaches can provide convincing mechanical sensations, they have inherent limitations in spatial precision and working distance.

We have been seeking alternatives using lasers as a means, attracted by the advantage that a laser allows its energy to be focused to a tight spot even across a long distance. In fact, lasers have been used as a stimulation tool in many studies of pain, mainly pertaining to the pricking or burning pain caused by laser-induced heat. The lasers used for the studies of pain typically had a pulse width longer than a few milliseconds and accompanied substantial increases in temperature^3^,^4^ for evoking the pains. For example, lasers causing temperature increase of 36 °C in 60 ms and 30 °C in 1.5 s were used for selective Aδ- and C-fiber nociceptor activation, respectively^3^. In contrast, we investigated the possibility of utilizing a laser for non-contact, painless haptic stimulation. Our results reported in this paper indicate that thermoelasticity induced by a laser can elicit tactile sensations with only a small increase in temperature, provided that the laser is applied with a low energy and a few-nanosecond pulse width in a linear optical regime. Specifically, we performed two physical experiments, *in vitro* and *in vivo*. In the former, we were able to detect the mechanical impact of a laser on cadaver skin samples that had optical and mechanical characteristics similar to those of the *in vivo* human skin. In the latter, we observed spatiotemporal temperature changes within an *in vivo* human skin using an infrared camera and confirmed that laser-induced temperature increases remained less than a few degrees. The temporal pattern of the temperature change was such that instantaneous increases in temperature were followed by an exponential decay due to the thermal diffusion. We further carried out a Monte Carlo simulation to estimate laser-induced temperature changes in the human skin and also simulated the thermoelastic wave equation to theoretically expect the thermoelastic effects on the human skin. The absolute value of the maximum temperature change was slightly different but comparable to that in the *in vivo* experiment, and the thermal diffusion time estimated from theory was in good agreement with that in the *in vivo* experiment. Lastly, human electroencephalography (EEG) experiments showed that brain responses to mechanical and laser stimuli were in good agreement, also supporting the idea that laser stimulation can induce tactile sensations.

## Mechanical effect of laser irradiation on a cadaver skin sample

The interaction of laser with matter and its application to many fields have been extensively investigated since the ruby laser was developed in 1960, thereby having a variety of high-power applications such as laser fusion, laser annealing, non-linear optics, and tissue ablation and coagulation in medicine, as well as low-power applications such as optical fiber communication and spectroscopy. Hitherto the mechanical aspect of laser interaction with matter has been known to be related to the generation of stress (or acoustic) waves based on five major interaction mechanisms: dielectric breakdown, vaporization or ablation, thermoelastic processes, electrostriction, and radiation pressure. In a linear-interaction regime with low-power radiation having a short pulse width of a few nanoseconds or less, the thermoelastic process is dominant, especially in interaction with a biological medium like tissue^5^,^6^,^7^,^8^. When the laser beam irradiates the tissue, the incident light produces a light energy distribution in the tissue by the incident light according to the optical properties of the skin such as absorption coefficient, scattering coefficient, and refractive index. The incident light energy is then transformed to thermal energy, reflecting the thermal properties of the tissue such as conductivity, heat capacity, convective coefficients, and emissivity, and temperature is increased in the tissue, followed by heat transfer to the surrounding tissues. In this case, two types of photo effects are involved according to the energy level and increasing rate of temperature: photo-thermal effect and photo-mechanical effect. In the photo-thermal effect, heat energy is accumulated enough to cause tissue coagulation or ablation with vaporization and pyrolysis. On the other hand, in the case of photo-mechanical effect, the instantaneous heating of tissue due to the energy absorption by pulsed-laser radiation induces rapid thermal expansion of the heated volume in tissue and produces thermoelastic waves as the heated volume reconfigures to a new equilibrium state. The thermoelastic waves appear as a transient waveform and propagate into the tissue at a sound speed of ~1.5 km/s, making a mechanical displacement in the tissue and thereby providing a possibility of activating mechano-receptors. It needs to notice that it is strain (or equivalently displacement), not stress, that directly causes the physical sensations, if any.

**Figure 1:**
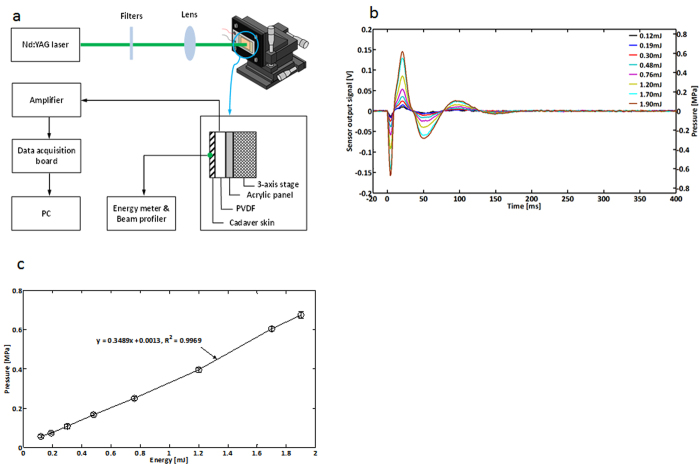
Physical experiments: Laser irradiation of the cadaver skin sample. **a**. A schematic diagram of the experimental setup to detect the mechanical impact of laser on the cadaver skin sample. The frequency-doubled Q-switched pulsed laser (5 ns pulse width) was used to generate a single-shot laser beam at a 532 nm wavelength. The generated beam was adjusted to focus on the skin sample through an optical filter and a lens. The spot size of the beam on the skin sample was 0.48 mm. The cadaver skin sample was attached to the front side of a PVDF transducer. Eight different beam energies used in the experiment ranged from 0.12 to 1.90 mJ, remaining under the MPE level to ensure no biological damage to the skin sample. **b**. The time series of the PVDF transducer sensor output signals recorded from 20 ms before to 400 ms after stimulation are represented in terms of voltage [V] along the left vertical axis and pressure [MPa] along the right vertical axis, respectively. Complex patterns of mechanical waves were consistently observed for eight different beam energy levels. The first negative peaks (~5 ms after stimulation) were generated by stress waves arriving at the sensor. The subsequent complex waveforms were attributed to the dynamic of the sensor and the measurement setup. **c**. Pressure values measured at the first negative peaks generated from the different beam energy levels. The open circles denote the average pressure values and the vertical bars denote the standard deviation. The maximum pressures were 0.68 ± 0.02 MPa generated by the highest beam energy (1.90 mJ). The pressure values were linearly correlated with the beam energy levels (*r*^2^ = 0.9969, the slope of the linear regression model = 0.35 MPa/mJ). (Fig. 1 was obtained from Konkuk University, drawn by MATLAB and MS power point software and created by Jae-Hoon Jun).

Figure 1a illustrates a schematic diagram of the experimental setup to detect the mechanical impact of laser on a cadaver skin sample that has optical and mechanical characteristics largely similar to those of *in vivo* human skin. A frequency-doubled Q-switched laser was used at 532 nm wavelength and 5 ns pulse width. The cadaver skin sample was attached to the front side of a polyvinylidence fluoride (PVDF) transducer. The spot diameter was set to be 0.48 mm. To check the energy dependence of the laser-induced mechanical effect, the pulsed laser was irradiated with beam energies varying from 0.12 to 1.90 mJ, which were under the maximum permissible exposure (MPE) level of 20 mJ/cm^2^ when averaged over a limiting aperture of 3.5 mm diameter, ensuring the safety of skin exposure to laser irradiation. A histological examination was then performed to verify that the mechanical effect of the laser irradiation was not the secondary phenomena due to damage to the cadaver skin. The stained sections showed typical morphological features of the normal thin skin in both the control and laser-irradiated cadaver skins (supplementary Figure 1).

In Fig. 1b, the average of temporally recorded output signals is presented in voltage [V] along the left vertical axis and in pressure [MPa] along the right vertical axis, respectively. A complex wave pattern of mechanical waves consistently occurred as the beam energy varied. The front negative peaks were created by stress waves arriving first at the sensor and the subsequent complex waveforms were attributed to the dynamic of the sensor and the measurement setup. The maximum pressures were observed when the beam energy was the highest (1.90 mJ). In Fig. 1c, the pressure produced by strain was plotted and its maximum value was 0.68 ± 0.02 MPa. A linear relationship existed between the pressure and the beam energy, having a slope of 0.35 MPa/mJ. In conclusion, it is confirmed that the mechanical effect is generated effectively when the pulsed laser irradiates the cadaver skin sample with low powers varying under the MPE level. Although this *in vitro* cadaver skin experiment had a boundary condition different from the *in vivo* human skin experiment, it provided a possibility of inducing tactile sensations in the skin, detecting the stress waves due to thermoelastic effects in the cadaver skin whose major component is collagen as is in the *in vivo* human skin.

### Infrared imaging of *in vivo* human skin

As rapid thermal expansion is closely related to skin deformation, the assessment of temperature changes in the human skin may reflect the occurrence of thermoelastic effects. We thus aimed to show *in vivo* the instantaneous and relatively small increase in temperature in the case of the thermoelastic effects with no thermal damage to the skin. We measured the laser-induced temperature change at the surface of the index finger using the infrared (IR) camera.

**Figure 2:**
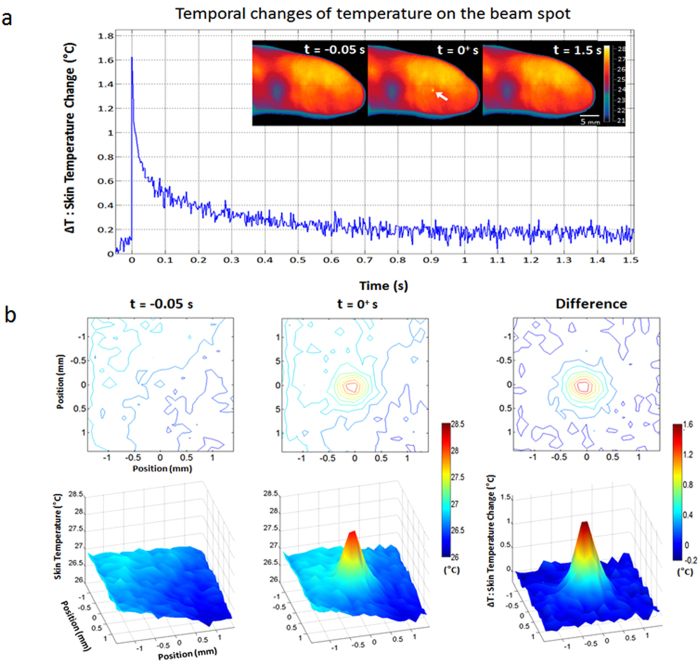
In vivo skin temperature measurements: laser-induced changes in temperature on the human skin surface measured by the IR camera. **a.** Temporal changes of the temperature at the beam spot of the index finger, measured from 0.05 s before to 1.5 s after laser stimulation. The same laser as in Fig. 1, the frequency-doubled Q-switched pulsed laser with a 532 nm wavelength and a 5 ns pulse width, was used. The IR images obtained at 0.05 s before a stimulus, the onset of a stimulus, and 1.5 s after a stimulus, are shown at the top-right corner of the plot, from left to right. The spatial resolution of the IR images was 0.14 × 0.14 mm^2^. The temporal resolution, or frame rates, was 400 Hz. The maximum increase in temperature was 1.63 °C. **b.** Spatial temperature distributions in the vicinity of the beam spot under *in vivo* condition. Both 2-D contour (upper row) and 3-D plots (bottom row) of spatial temperature distributions were provided. The first and second columns represent the spatial temperature distributions before and after laser stimulation, respectively. The last column shows the spatial distribution of the temperature change (Δ*T*) obtained by subtracting the first column from the second column. (Fig. 2 was obtained from Konkuk University, drawn by MATLAB software and created by Jae-Hoon Jun)

The plot in Fig. 2a shows the temporal changes of temperature at the beam spot in response to laser stimulation with energy of 1.90 mJ and a pulse width of 5 ns. IR imaging was conducted from 0.05 s before stimulation to 1.5 s after stimulation at a frame rate of 400 Hz. The spatial resolution of IR images was 0.14 × 0.14 mm^2^. The temporal profile demonstrated that energy absorption at the onset of laser stimulation resulted in an instant increase in temperature by 1.63 °C. The temperature then exponentially decayed over time with a time constant of 60 ms, returning to its original state. The observed time constant (60 ms) was on a par with the theoretically expected time constant (on the order of 100 ms) in the thermal diffusion mechanism. Temperature change observed in the *in vivo* experiment was 0.87 °C lower than that in the simulation study, which may be attributed to the limits in the spatial and temporal resolutions of the IR camera as well as individual variations in optical coefficients.

**Figure 3:**
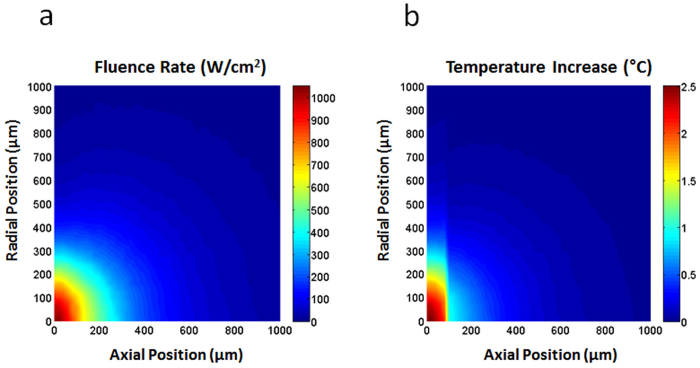
Monte Carlo simulation results: Distributions of the fluence rate (**a**) and temperature increases (**b**) in the skin. **a.** The fluence rate distribution in the skin obtained from the Monte Carlo simulation with an incident laser power of 1 W. The spatial profile of the incident light was assumed to follow the Gaussian distribution with a 1/*e* diameter of 0.48 mm. **b.** The temperature increase distribution in the skin calculated with a pulse energy of 1.90 mJ. The skin was assumed to be composed of two layers (epidermis and dermis). The maximum temperature increase was ~2.5 °C and the effective diameter at the 1/*e* level was ~0.55 mm. (Fig. 3 was obtained from Chosun University, drawn by MATLAB software and created by Jong-Rak Park)

Figure 2b shows the spatial temperature distribution near the beam spot on the index finger, distributed around the beam spot. Both 2-D contour and 3-D plots of spatial temperature distributions were provided for better representation. The first and second columns represent the spatial temperature distributions before and after laser stimulation, respectively. The last column shows the spatial distribution of the temperature change (ΔT) that was obtained by subtracting the first column from the second column. The effective diameter of the heated region was estimated to be ~0.59 mm at the 1/e level by averaging those determined at four different directions, which was comparable to the result of the Monte Carlo simulation considering the scattering effects, i.e., 0.55 mm, given in the next section (Fig. 3b).

In sum, the *in vivo* results support the possible generation of strain waves due to thermoelastic effects of laser stimulation, showing rapid increases in the human skin temperature by 1.63 °C with a laser stimulus having a 5 ns pulse width and a 1.90 mJ energy. A more desirable solution for verifying the strain waves generated by laser stimuli might be the direct measurement of the physical displacement of human skin *in vivo*, e.g., by using an optical microscope or an interferometer. From the simulation given in the following section, temperature increases by ~2 °C is expected to produce a displacement of the human skin in the order of a few-hundred nanometers. In this case, an optical microscope is not available in terms of spatial resolution, whereas an interferometer that enables the measurement of nano-scale displacements might be regarded as a good alternative. However, it is also intractable in practice to use the interferometer for measuring such a small displacement of the *in vivo* human skin since it can be substantially interfered by involuntary body movement that is likely to generate unexpected displacement. Therefore, we instead estimated the possible displacement of human skin due to laser stimulation by using the Monte Carlo simulation and subsequent simulations of the thermoelastic wave equation, which will be described in the following section.

### Simulations of laser-induced thermoelastic effects in human skin

We also performed Monte Carlo simulations to properly take into account the light transport and absorption characteristics in human skin. For simulation, skin was assumed to have two layers: epidermis and dermis. The thickness of epidermis was set to be 100 μm, which is the average value of the epidermal thickness reported by the previous study using optical coherence tomography imaging^9^. The wavelength and spatial profile were assumed to be 532 nm and Gaussian with a 1/*e* diameter of 0.48 mm, respectively. The same parameters setups, including the pulse energy used for the calculation of temperature increase described shortly, were also applied for the study of brain responses to laser stimulation using human EEG experiments which will be discussed later.

Figure 3a shows the fluence rate distribution in the skin obtained from the Monte Carlo simulation when the incident laser power was 1 W. Based on this result, the distribution of temperature increase (see Fig. 3b) was also calculated according to^10^


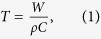


where *W* is the distribution of absorbed energy density, *ρ* is the mass density, and *C* is the specific heat. The pulse energy of the incident light was assumed to be 1.90 mJ. The optical and physical properties of human skin were adopted from the recently published literatures^11^,^12^ and were presented in the Method Section in detail. Because of the difference in the absorption coefficients between the epidermis and dermis layers, the temperature distribution showed an abrupt change at the boundary between the two layers. The maximum increase in temperature was ~2.5 °C near the skin surface. The effective diameter at the 1/*e* level was measured to be ~0.55 mm.

**Figure 4:**
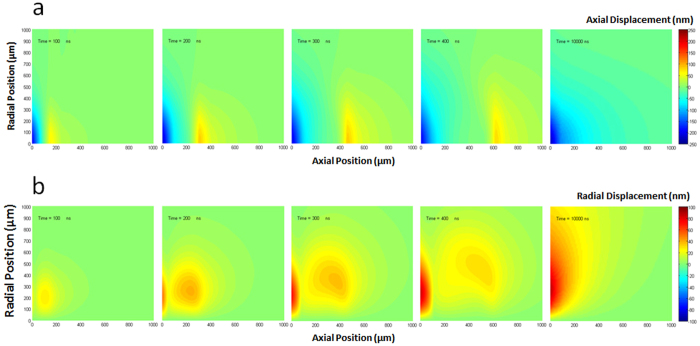
Simulation results of the thermoelastic wave equation: Axial displacement *u_z_* (**a**) and radial displacement *u*_*r*_ (**b**) at 5 different time points (100 ns, 200 ns, 300 ns, 400 ns, and 10000 ns) after the incidence of a 5 ns laser pulse. **a.** The maximum amplitudes of axial displacement were ~85 nm for the transient waves and ~230 nm for the quasi-steady-state solutions, respectively. Positive values indicate displacement into the skin and negative toward the air. **b.** The maximum amplitudes of radial displacement were ~40 nm for the transient waves and ~90 nm for the quasi-steady-state solutions, respectively. For both cases, the transient waves propagate into the skin at a speed of sound with decreasing amplitudes and the quasi-steady-state is reached at 10,000 ns in a 1000 μm (axial) × 1000 μm (radial) region under the skin surface. (Fig. 4 was obtained from Chosun University, drawn by MATLAB software and created by Jong-Rak Park)

Next, we simulated the thermoelastic effects induced by a pulsed laser on human skin by numerically integrating the thermoelastic wave equation^13^,^14^, which was summarized in the Method Section, for the temperature distribution obtained from the Monte Carlo simulation. Temperature was assumed to linearly increase during the laser-pulse duration (i.e., 5 ns). Assuming the symmetry in the radial direction, cylindrical coordinates were adopted, where the *z*- and *r*-axes represent the direction into the skin (axial direction) and the radial direction, respectively. Figure 4a,b show the simulation results of the axial (*u*_*z*_) and radial (*u*_*r*_) components of the displacement vector **u** that fully defines the deformation of the skin as a function of time. The spatial distribution of *u*_*z*_ and *u*_*r*_ were presented at five different times (100 ns, 200 ns, 300 ns, 400 ns, and 10,000 ns) after the incidence of the laser pulse. It was seen that *u*_*z*_ and *u*_*r*_ gradually build up to a considerable extent near the skin surface while parts of them propagate into the skin. In fact, it has been known that the solution of the thermoelastic wave equation is divided into two groups: the time-dependent transient solution and the time-independent quasi-steady-state solution^15^. The transient solution propagates at the speed of sound and ends up disappearing in the region near the skin surface. In contrast, the quasi-steady-state solution emerges near the skin surface after the transient solution decays away. According to our simulation, it was observed that the amplitude of the transient solution reached the maximum between 200 ns and 300 ns and then kept decreasing while it was travelling into the skin. Since the quasi-steady-state solution is caused by the non-uniform temperature distribution, it also gradually disappears with thermal diffusion^15^. We found no essential change after 10,000 ns in the region of a 1 mm (axial) × 1 mm (radial). The maximum amplitudes of the transient solution were ~85 nm for *u*_*z*_ and ~40 nm for *u*_*r*_, respectively, whereas they were ~230 nm for *u*_*z*_ and ~90 nm for 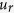, respectively, in the case of the quasi-steady-state solution.

Since thermoelastic waves have a spatial extent on the order of the dimension of the heated region and they propagate at the speed of sound, the temporal duration *τ*_*str*_ for which the transient thermoelastic waves continue to exist in a certain region of the skin is given by^10^


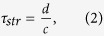


where *d* is the characteristic length of the heated region and *c* is the speed of sound in the skin (≈ 1540 m/s). *τ_str_* is also known as the stress confinement time. Due to the larger absorption coefficient of the epidermis layer, *d* can be assumed to be the thickness of the epidermis layer, i.e., ~100 μm. In this case, *τ_str_* = 65 ns. On the other hand, the characteristic time scale for the decay of the quasi-steady-state solution, which is comparable to the thermal diffusion time, can be estimated by^16^


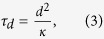


where *κ* is the thermal diffusivity. For a typical value of *κ* (= 0.15 mm^2^/s) for a biological tissue^17^, τ_d_ = 67 ms according to Eq. (3). This characteristic time is on the order of 100 ms. Although the amplitudes of the transient solution are smaller than those of the quasi-steady-state solution and τ_str_ is far smaller than τ_d_, it is probable that the transient thermoelastic waves play a major role in the sensory-evoked response by causing a sudden mechanical blast that can activate all sensory receptors near the skin surface almost at the same time with the help of their unique dynamic characteristics.

### Behavioral and electroencephalography (EEG) experiments

Lastly, a similarity between mechanical and laser stimulations was demonstrated in terms of behavioral and cortical responses in humans. For cortical responses, we focused on the characteristics of cortical activity that can be noninvasively measured via EEG or magnetoencephalography (MEG) and have been examined for comprehending how somatosensory information is processed in the brain^18^,^19^. It has been demonstrated that both innocuous and noxious stimuli induce changes in the amplitude and phase of cortical oscillations^18^. In particular, an innocuous tactile stimulus elicits decreases in the spectral power of EEG in the alpha-frequency band (11–13 Hz) and the beta-frequency band (21–28 Hz), followed by a subsequent increase in the beta-frequency band, over the sensorimotor cortical area^18^,^19^,^20^,^21^. In EEG, this type of decrease and increase in the power of cortical oscillations are often called event-related desynchronization (ERD) and synchronization (ERS), respectively. Noxious heat and laser stimuli also generate ERD and/or ERS in the alpha and beta band over the sensorimotor area^22^,^23^. In the present study, we investigated how these alpha and beta ERD/ERS patterns varied with different stimuli including mechanical pressure, laser and heat. We hypothesize here that, if one feels a non-nociceptive tactile sense by laser stimulation, the induced ERD/ERS patterns in this case should be similar to those by mechanical tactile stimulation but different from those by nociceptive laser stimulation. In addition to the ERD/ERS analysis, we also investigated the latency of event-related potentials (ERPs) induced by different stimuli. Since sensory input mediated by cutaneous nociceptive fibers reaches the cortical areas more slowly than that mediated by non-nociceptive fibers does, we assumed that the latency of ERPs induced by nociceptive laser stimulation would be longer than that induced by non-nociceptive laser stimulation.

**Figure 5:**
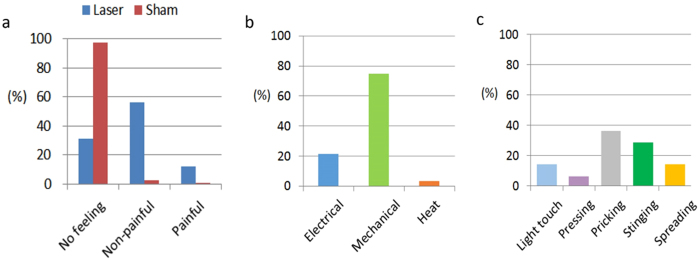
Human tactile sensation experiments: Behavioral responses to laser stimuli. **a.** Behavioral responses of twenty participants to laser stimulation with intermittent sham stimuli. Participants were asked to choose one of three feelings, i.e., none, non-nociceptive, and nociceptive, after each stimulus. They could tell the presence of laser stimulation from its absence with an accuracy of 75.8%. The sensitivity (feeling for laser stimuli) and the specificity (no feeling for sham stimuli) were 68.6% and 97.4%, respectively. With laser stimulus, 56.3% of them reported a non-nociceptive feeling and 12.3% of them a nociceptive feeling, and 31.4% of them with no feeling. **b.** Classification of the post-session subjective description of tactile sensations by the participants who reported non-painful feeling in response to laser stimuli. The subjective descriptions were classified as mechanical sensations (75%), electrical (21.4%) or heat (3.6%). **c.** A further distribution of the descriptions corresponding to mechanical sensations: light touch (14.3%), pressing (6.3%), pricking (36.5%), stinging (28.6%), and spreading (14.3%). It indicates that regular mechanical sensations (light touch and pressing) were perceived with a rate of 20.6%, while those associated with a very narrow contact area (pricking and stinging) were elicited with a rate of 65.1%. (Fig. 5 was obtained from Pohang University of Science and Technology, drawn by MATLAB software and created by Seungmoon Choi)

We first examined the behavioral responses of participants to laser stimulation also by delivering sham stimuli intermittently. After each stimulation, the participants were asked to choose one of the three answers: no sensation, non-painful sensation, and painful sensation. They detected the laser stimulation with an accuracy of 75.8%. The sensitivity of perceiving a stimulus given the laser stimulus was 68.6%, and the specificity of perceiving no stimulus given the sham stimulus was 97.4%. Furthermore, when the laser stimulus was presented, 56.3%, 12.3%, and 31.4% of the participants reported a non-painful sensation, a painful sensation, and no sensation, respectively (Fig. 5a). It is noted that to avoid any skin damage, laser parameters were chosen to be within the MPE level. After each experiment, we visually confirmed that no damages were made to the irradiated skin area for each participant. The result that 12.3% of the participants perceived pain from the laser stimulation is likely due to individual differences in uncontrolled factors such as gender^24^,^25^, age^26^,^27^, and adipose thickness^28^.

The subjective descriptions of the participants who reported non-painful sensations were classified as more mechanical sensations (75%) than electrical (21.4%) or heat (3.6%), as shown in Fig. 5b. The descriptions that corresponded to mechanical sensations were further distributed as (Fig. 5c): light touch (14.3%), pressing (6.3%), pricking (36.5%), stinging (28.6%), and spreading (14.3%). Regular mechanical sensations (light touch and pressing) were elicited with a rate of 20.6%, while those associated with a very narrow contact area (pricking and stinging) were perceived with a rate of 65.1%. Spreading is a unique sensation to laser stimulation, and it is often described by the participants as a sensation that begins to feel at a point just below the skin and then soon progresses into the skin with an increasing volume. The grounds for spreading are unknown at the moment, but one possible explanation is that a sudden thermoelastic blast induced by laser stimulation might bring about the sensation by activating all sensory receptors in the vicinity nearly at the same time.

Lastly, we examined the response time (RT) of participants in response to laser stimulation. For each response of no sensation, non-painful sensation, and painful sensation to laser stimulation, the average RT was 606 ± 191 ms, 460 ± 151 ms, and 513 ± 152 ms, respectively. A *t*-test showed that the average RT for non-painful sensation was significantly shorter than that for painful sensation (*p* < 0.05). This implies that the laser-induced non-painful sensation was likely to be delivered through non-nociceptive Aβ-fiber afferents which are faster than nociceptive afferents. It is also speculated that the longer RT for no sensation might be due to additional cognitive processes in participants ensuring that no stimulus was detected.

**Figure 6:**
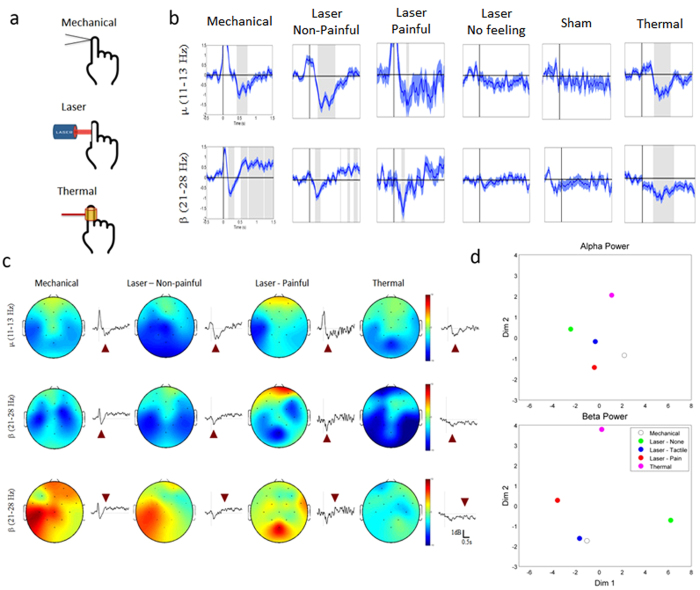
Human EEG experiments: Cortical responses to mechanical, laser, and thermal stimuli. **a.** Simple illustrations of mechanical, laser, and thermal stimulations. **b.** Temporal power change of cortical oscillations evoked in the contralateral sensorimotor area by pressure, laser, and thermal stimuli. Post-stimulus periods with significant power changes (*p* < 0.01) were marked as shaded areas. Mechanical stimulation induced the alpha (420 ~ 839 ms) and the beta ERD (212 ~ 316 ms), as well as the beta ERS (526 ~ 1,500 ms). Thermal stimulation induced the alpha (354 ~ 907 ms) and the beta ERD (395 ~ 599 ms) without beta ERS. No significant change was observed when participants reported no feeling by laser stimulation or when the sham stimulus was given. The alpha (379 ~ 442 ms) and beta ERD (233 ~ 316 ms) were found when participants reported nociceptive feelings by laser stimulation. When participants reported non-nociceptive feelings, the beta ERS was observed from 923 ms after stimulus ons*et al*ong with the alpha (254 ~ 756 ms) and beta ERD (169 ~ 316 ms). **c.** Brain topography was shown for the EEG data selected at a specific time (indicated by triangles) when the change of ERD/ERS was most distinct. All the stimuli induced the alpha ERD (first row) and beta ERD (second row) over the contralateral sensorimotor area (first row). Following the beta ERD, the sensorimotor beta ERS was generated only by the mechanical and non-nociceptive laser stimuli (third row). In addition, the posterior beta ERS was observed in the middle posterior area by nociceptive laser stimuli. **d.** 2-D spatial representation of the cortical oscillations. Spatio-temporal ERD/ERS patterns responding to different stimuli were projected onto a 2-D space using multidimensional scaling. In alpha oscillations, ERD/ERS patterns with laser-induced non-nociceptive and nociceptive feelings were closer to those with mechanical stimulation. In beta oscillations, ERD/ERS pattern with laser-induced non-nociceptive feeling was much closer to mechanical senses than others. (Fig. 6 was obtained from Ulsan National Institute of Science and Technology, drawn by MATLAB software and created by Sung-Phil Kim)

Figure 6b shows the temporal power change of cortical oscillations evoked in the contralateral sensorimotor area by different stimuli such as pressure, laser, and heat. Post-stimulus periods showing a significant power change with respect to the baseline (i.e., the average power before stimulus onset) were marked as shaded areas, using the statistical evaluation based on a *t*-test (*p* < 0.01). While the mechanical stimulation induced the alpha ERD and the beta ERD as well as the beta ERS, the thermal stimulation induced the alpha ERD and the beta ERD, but no significant beta ERS. Cortical responses to the laser stimulation were analyzed in conformance with the behavioral responses of the participants. First of all, no significant alpha ERD, beta ERD, and beta ERS were observed for the participants who reported no feeling. The sham stimulus also induced no significant alpha or beta ERD/ERS. However, the alpha ERD and the beta ERD were found over the contralateral sensorimotor area when they reported a nociceptive feeling. On the other hand, when they reported an innocuous feeling, the beta ERS was also observed along with the alpha ERD and the beta ERD. Therefore, the results of the alpha and beta ERD/ERS analyses indicate a similarity in cortical activity patterns between the mechanical stimulation and the laser stimulation with innocuous feelings.

To visually represent the cortical responses over the brain, brain topography is presented in Fig. 6c using the data selected at a specific time (indicated by a purple triangle) when the variation of cortical oscillations was most distinct. All of the stimuli evoked the alpha ERD over the contralateral sensorimotor area (a first row). The beta ERD was also induced by all the stimulations with a latency of 500 ms (a second row) and, after the beta ERD disappeared, the beta ERS was generated in the sensorimotor area only by mechanical and laser stimulations with innocuous feelings (a third row). It is noteworthy that, when the laser stimulation was reported to cause a noxious feeling, the beta ERS was observed in the middle posterior area and not in the contralateral sensorimotor area^29^.

To better demonstrate the similarity between mechanical and innocuous laser stimulations in comparison to other stimulation techniques, similarities between cortical power-change patterns responding to different stimuli were calculated by linear correlation and were projected onto a 2-dimensional space using multidimensional scaling (MDS), as shown in Fig. 6d. In the case of the alpha oscillations, laser stimulation with a non-nociceptive or nociceptive feeling was spatially closer to mechanical stimulation than thermal or laser stimulation with no feeling. On the other hand, in the case of beta oscillations, laser stimulation with a non-nociceptive feeling was far closer to mechanical stimulation than others, thus demonstrating that laser stimulation with a non-nociceptive feeling can elicit cortical responses similar to innocuous mechanical stimulation.

Finally, we measured the latencies of the ERPs in response to laser stimulations by measuring the latencies of the first large negative peaks of the ERPs. When participants reported a noxious feeling, the average latency was 236 ± 34 ms. In contrast, when they reported an innocuous feeling, the average latency was 165 ± 46 ms. We also measured the latency of the ERPs in response to mechanical simulations and found the average latency to be 152 ± 23 ms. These results conformed with those of the laser-evoked potential (LEP) elicited at 220 ms (N220) with painful sensation or those of the somatosensory-evoked potential (SEP) elicited at 140 ms (N140) with non-painful tactile sensation^30^,^31^. A significant difference in latency was found between mechanical, painful laser and non-painful laser stimulations (p < 0.01). Post-hoc *t*-tests revealed a difference between painful and non-painful laser stimulations or between painful laser and mechanical stimulations (p < 0.01, Bonferroni correction), but not between non-painful laser and mechanical stimulations (p > 0.05, Bonferroni correction). It is thus suggested that cortical responses to the laser stimulation generating an innocuous feeling are likely to be induced by non-nociceptive fast input, whereas those to the laser stimulation generating a noxious feeling are induced by nociceptive slow input.

## Discussion

In this paper, we have investigated the possibility of utilizing laser for innocuous tactile stimulation and supported it by simulation and experiments. In the *in vitro* experiment, we showed that the laser stimulation with low power and a short pulse width of 5 ns can produce an innocuous mechanical effect on the cadaver skin sample that has optical and mechanical characteristics largely similar to those of *in vivo* human skin. Then, we performed IR imaging of the *in vivo* human skin to measure the temperature increases under *in vivo* condition, which was less than 2 °C. Next, we performed the Monte Carlo simulation to confirm the small increases in temperature by simulation (~2.5 °C) and also simulated the thermoelastic wave equation to theoretically expect the laser-induced thermoelastic effects on the human skin, thereby finding a transient solution that explains the transient displacement propagating into the skin. Lastly, at the perceptual level, we presented the EEG responses to the laser stimulation in comparison with other stimulations including the mechanical stimulation. As demonstrated in cortical oscillations, brain topography, and a 2-D similarity space, the laser and mechanical stimulations showed a good degree of similarity in their EEG responses.

Although the existence of a mechanical effect in laser interaction with matter has already been known and its mechanism has been well elucidated, to the authors’ knowledge, its application in tactile stimulation has not been suggested yet. Especially, the laser-induced mechanical effects on tissues have found their applications mainly in medicine, utilizing the mechanical aspect of highly-powered laser interaction with matter such as evaporation and ablation, or in the study of pain. Despite the controversial nature of laser stimulation for research on pain, a number of studies of pain reported advantages of using laser as an accurate and selective stimulation means^3^,^4^,^33^,^34^. The previous studies of pain using laser stimulation primarily evoked heat-induced pain as laser stimuli were used to cause drastic increases in temperature (usually >20 °C)^3^,^4^. However, both our simulation study and the IR imaging of the *in vivo* human skin showed that temperature was likely to increase as much as 2.5 °C with a short pulse width and a low energy level. Such a small temperature change may imply that laser stimulation in our study could induce mechanical sensations based on the thermoelastic effects, different from nociceptive sensations induced by heat. Although it is possible that the step increases in skin temperature by ~2.5 °C due to the laser stimulus could activate “low-threshold, innocuous” warm receptors such as C warm receptors^35^, we have observed only 3.6% of the subjects who reported the feeling of heat by the laser in the behavioral studies. In contrast, 75% of subjects reported the mechanical sensation, implying that the major effect of laser stimulation in our study was the mechanical one. Moreover, in the subjects who reported the mechanical sensation, the analysis of EEG pattern, reaction time (RT), and latencies of ERPs indicated that Aβ afferents were responsible for the sense. Taken together, it is suggested that laser stimulation in the present study evoked tactile sensation through a photo-mechanical mechanism, although the laser can also induce the sense of innocuous heat through warm receptors by small increases in skin temperature in a minor population of subjects. Further study is needed to investigate a possibility of selectively evoking tactile sensation without innocuous heat sensation. Our study explored only a limited range of laser parameters. In future studies, however, we expect to selectively stimulate single mechanoreceptors by tuning laser parameters as are the cases where heat-based laser was used to selectively stimulate the Aδ-fiber or C-fiber by tuning its parameters (power, stimulation duration, etc.)^3^,^32^,^36^. In particular, it would be possible to find the laser energy levels below the threshold of nociceptive receptors but above that of mechanoreceptors since the threshold of nociceptive receptors is known to be higher than that of mechanoreceptors and use that level of energy with an appropriate parameter setting to evoke various mechanosensations.

The merits of laser stimulation such as no need for contact, high spatial precision, and a wide range of working distances, are expected to manifest themselves in a variety of scientific or practical applications. For example, laser stimulation is likely to contribute to perception, neurophysiology, or brain research as an accurate tactile stimulator and function in interactive applications as a new natural, unobtrusive information-transmission modality.

From this viewpoint, our study has elements of novelty that could be useful for applications in the study of tactile sensation such as light touch^37^,^38^,^39^ since the study of tactile sensation requires highly specific control on the stimulus without pain or damage. For example, laser stimulation can be a good alternative to the way of an uncertain poking with glass pipettes for activating *in vitro* mechanosensing cells very specifically^37^,^39^,^40^,^41^. In support of this, we could evoke the non-selective cation currents, whose characteristics (single-channel conductance of ~30 pS, non-selective cation-permeability, and block by gadolinium and ruthenium red) are similar to those of Piezo ion channels^40^,^41^, in mechanosensing neuroblastoma N2A and Merkel cells (unpublished observation).

In this study, we did not directly measure the laser-induced displacement of the *in vivo* human skin, just offering the simulation data for it besides the IR imaging of the *in vivo* human skin to estimate the temperature change in the *in vivo* skin due to laser stimulation. Further studies are warranted that explore the state-of-the-art technology enabling the direct measurement of the mechanical impact to the *in vivo* human skin.

According to our study of the behavioral responses to laser stimulation, non-painful sensations other than mechanical sensations, such as electrical and heat sensations, were reported, though a majority of participants (75%) reported the mechanical sensations. Even in the mechanical sensations, however, such sensations as pricking (36.5%), stinging (28.6%), and spreading (14.3%), which are typically not regarded as the general mechanical sensations, were also reported. These results tell us that even more studies still need to be conducted to elaborately render tactile sensation by laser stimulation. A possible way is to tune the laser parameters such as wavelength and repetition rates etc., in addition to power, so that a specific parameter setting can induce a specific type of sensation. Since the displacement induced by thermoelastic effects is proportional to both temperature increase and the penetration depth of light^14^, laser pulses at different wavelengths could induce different amounts of displacements while maintaining the same temperature increase. Hence, alterations of the wavelength might be expected to provide a means of controlling the magnitude of the displacement, while avoiding the change of the temperature increase that would happen in regulating only the laser power. In view of the fact that most tactile sensations are the human responses to spatio-temporal stimuli from the environments, we also expect that modulations of the laser stimulation in both space and time would be ultimately necessary in order to induce intricate sensations such as tickling and vibration and so on.

Another possible way is to address this issue from the standpoint of neurophysiology. For example, as mentioned earlier, the feeling of spreading in mechanical sensations may be associated with a possibility that laser-induced thermoelastic effects indiscriminately and almost simultaneously activate all the sensory receptors in the vicinity. Because the previous researchers succeeded in selectively evoking pricking (Aδ-fiber) or burning (C-fiber) pain by tuning the protocol of laser stimulation^3^,^4^,^33^,^34^,^32^,^36^, further studies on the selective stimulations of somatosensory cells or sensory nerve endings by laser would shed light on the diverse characters of laser-induced tactile sensation. In parallel, other studies are also warranted that search for neurophysiological evidences to verify that laser can selectively stimulate tactile mechanoreceptors without activation of nociceptors.

## Methods

All examinations were performed under and approved by the regulations of Institutional Review Committee of Konkuk University Hospital (KUH 1160062) and Chosun University (IRB-13-008) and all methods were carried out in accordance with the approved guidelines.

### Mechanical effect of laser irradiation on a cadaver skin sample

#### Experimental setup

The frequency-doubled Q-switched laser (Brilliant B, Quantel, Santa Clara, CA, USA) was used with 532 nm wavelength and 5 ns pulse width. The generated pulsed beam was designed to focus on the cadaver skin sample that was attached to the front side of a polyvinylidence fluoride (PVDF) transducer (LDT1-028 K, Measurement Specialties, Hampton, VA, USA), passing through optical filters and a lens^5^. The spot diameter was set to be 0.48 mm by controlling the plano-convex lens (*f* = 250 mm) and 3-axis translation stage. We used eight different beam energies varying from 0.12 to 1.90 mJ, i.e., 0.12, 0.19, 0.30, 0.48, 0.76, 1.20, 1.70, and 1.90 mJ, by combining absorptive neutral density filters in various ways. The beam energy was measured with a pyroelectric energy detector (818E-05-12-L, Newport, Irvine, CA, USA) and an energy meter (1918-R, Newport, Irvine, CA, USA). The spot diameter was measured with a beam profiler (SP620U, Ophir-Spiricon LLC., N. Logan, UT, USA).

#### Maximum Permissible Exposure (MPE)

The MPE level represents the maximum energy level of laser radiation to which an unprotected person may be exposed without adverse biological damages to the eye or skin^42^. For the laser parameters used for this study, i.e., the wavelength of 532 nm and pulse width of 5 ns, the MPE level was 20 mJ/cm^2^ according to the following equation: *MPE* = 2*C_A_* × 10^−2^ [ J/cm^2^], where the wavelength correction factor *C_A_* is 1 in our case. For wavelengths between 400 nm and 1,400 nm, the MPE level is measured over a limiting aperture of 3.5 mm diameter, not being adjusted to consider smaller pupil diameters^43^. Since the energy density (ED) over the limiting aperture for specific laser energy and spot size is given by 


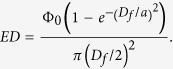


where *Φ*_0_ is the laser beam energy, *D_f_* is the limiting aperture diameter at skin and is set to be 3.5 mm, and *a* is the spot size and was 0.48 mm for this study, the energy densities calculated for the eight beam energies used for our study from 0.12 to 1.90 mJ are to be in the range of 1.26 ~ 19.76 mJ/cm^2^, which do not exceed the MPE level of 20 mJ/cm^2^.

#### Cadaver skin sample

This study was approved by the Institutional Review Board of Konkuk University Hospital (KUH1160062). The cadaver skin sample was extracted from the thigh of a 55-year-old male corpse and consisted of epidermis and dermis with subcutaneous fat removed. It has dimensions of 25 × 25 mm^2^, having thickness of 1.76 ± 0.11 mm. The surface of the epidermis was slick and the other surface of the dermis was rough with subcutaneous fat removed. The cadaver skin sample was preserved in formalin and washed in distilled water before experiments.

#### Signal acquisition and processing

The PVDF sensor had dimensions of 41.40 × 16.26 mm^2^ and was made up of a piezoelectric PVDF polymer film with 28 μm thickness laminated onto 0.125 mm thickness polyester. The sensitivity of the PVDF film was 13 mV/N. Output signals from the sensor were amplified by 40 dB and stored in PC using a data acquisition board (NI USB-6361, National Instruments, Austin, TX, USA) and LabVIEW (NI LabVIEW 2012, National Instruments, Austin, TX, USA). By considering the sensitivity of the PVDF film, amplification gain, and the spot size of laser irradiation, the sensitivity of the PVDF transducer was recalculated in terms of pressure, i.e., 235.2 mV/MPa, and used to convert the output signals in mV to MPa. One hundred one-pulse single shots were repeatedly performed at each energy level and output signals were averaged for improving the signal-to-noise ratio (SNR). Any damage to the sample was checked every ten single shots using a digital microscope (AM3713TB, AnMo Electronics Corp., Taiwan). Output signals were processed with home-built programs based on MATLAB (R2008a, Mathworks, Portola Valley, CA, USA).

### Infrared imaging of *in vivo* human skin

To examine the laser-induced thermoelastic effects in the skin, we measured the spatial and temporal profiles of the temperature changes in the *in vivo* human skin using a high-speed infrared (IR) imaging camera (X6540sc, FLIR Systems, Inc., Wilsonville, OR, USA), with an image matrix size of 320 × 256, a 400 Hz frame rate, and a 0.02 °C temperature resolution. The IR imaging shared the same experimental setup as the cadaver skin and human EEG experiments: the energy level of laser was 1.90 mJ, the beam diameter was 0.48 mm, and the room temperature was 17.3 °C. These parameters met the MPE safety criterion. The temperature distribution in the skin was extracted frm the IR images using the FLIR software (FLIR Systems, Inc., Wilsonville, OR, USA) and visualized using the MATLAB software. Note that the IR imaging could detect the temperature changes with no physical contact to the skin, avoiding any contact-related heat loss and making it possible to measure subtle temperature changes.

### Simulations of laser-induced thermoelastic effects in human skin

#### Monte Carlo simulation

Because of the strong light scattering in biological tissues, an analysis of laser radiation transport that includes the calculation of temperature increases distributed over the human skin caused by light absorption is not straightforward. We employed an optical simulation tool, TracePro (Lambda Research Corporation, Littleton, MA, USA), which is capable of simulating light transport in biological tissues. In this simulation study, optical properties of the human skin at 532 nm wavelength, including refractive index *n*, anisotropy factor *g*, absorption coefficient *μ*_a_, and scattering coefficient *μ*_s_, were selected from a recent report^11^ and are listed in Table 1.

#### Simulation of the thermoelastic wave equation

The thermoelastic wave equation is given by^13^,^14^


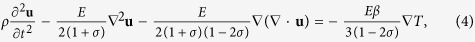


where *ρ*, **u**, *E*, *σ*, *β*, and *T* represent mass density, displacement vector, Young’s modulus, Poisson’s ratio, volumetric thermal expansion coefficient, and distribution of temperature increase, respectively. From Eq. (4), one can easily find that the driving term for the displacement vector, which completely defines the deformation of an elastic medium as a function of time, is the gradient of the temperature distribution. Non-uniform temperature increases are, therefore, essential for the generation of thermoelastic waves. Here, note also that when the temperature distribution has zero gradients, Eq. (4) can be reduced to the ordinary wave equations with the longitudinal (*c*_*l*_) and transverse (*c*_*t*_) speeds of sound expressed in terms of *ρ*, *E*, and *σ* by^13^


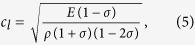



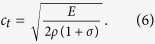


Due to the symmetry of the problem, cylindrical coordinates are adopted. Hence, the displacement vector **u** has the axial displacement *u*_*z*_ and the radial displacement *u_r_*. The thermoelastic wave equation was integrated using the Adams-Bashforth time-stepping method and the simulation code was programmed using MATLAB software.

Typical values for the physical properties of the human skin, including mass density *ρ*, specific heat *C*, Poisson’s ratio *σ*, volumetric thermal expansion coefficient *β*, and longitudinal speed of sound *c*_*l*_, were also chosen from recent studies^12^,^44^,^45^,^46^ and are listed in Table 2.

### Behavioral and EEG experiments

#### Participants

Twenty-one adults (7 female, mean age 22.4 ± 2.4 years) participated in this study. Nineteen of them were right-handed, and none of them had neurological or neuropsychological disorder according to their self-report. The Institutional Review Board at Chosun University approved the study (IRB-13-008). All participants signed on a written consent after they had been well informed of the whole procedure of the study.

#### Stimuli

Mechanical stimuli were provided by a custom pressing device. The device generated mechanical pressure through the rotation of a wooden stick (2 × 3 mm^2^ cross-section) via an electric motor that resulted in the translational motion of the stick parallel to the axis of rotation. The 4.35° rotation of the wooden stick provided the pressure stimulation with 1.26 × 10^−3^ psi, making a 4 mm depth of indentation. Heat stimuli were delivered by another device that transferred heat onto the skin through air to avoid the compounding effects of mechanical contact. The device had a cylindrical coil that dissipated heat with electric current, and participant’s index fingertip was placed at the center of the coil. The coil diameter was 40 mm, and the distance between the fingertip and the coil was approximately 10 mm. The temperature of the heat stimuli and the baseline was 42 °C and 23 °C, respectively, and the rising time needed to reach the specified temperature from the baseline was 3 s. For laser stimulation, the pulsed laser with 0.48 mm spot size and 1.90 mJ beam energy was irradiated on the index fingertip in the same experimental setup as the experiment using the cadaver skin sample.

#### Tasks

Each participant was presented with sixty mechanical stimuli, sixty laser stimuli, and sixty heat stimuli to the index finger. The experiment consisted of three respective sessions for laser, mechanical, and heat stimulations with a 10 min inter-session break. In the laser-stimulation session, each trial started with a resting period of 19 s, followed by an auditory instruction (a pre-recorded speech meaning “get ready’) given to indicate an upcoming stimulus in 4 s. Then, a single-shot laser stimulus was irradiated on the right index fingertip. A high-frequency beep was given 3 s after the stimulus onset to guide participants to begin a self-rating of the feel of the laser stimulation. After 4 s, another auditory instruction (a pre-recorded speech for “have a rest”) was issued to indicate the end of the current trial and also the start of the next trial with a resting period of 19 s. The laser stimulus was repeated 60 times for each participant. A sham stimulus that included only the triggering sound of the laser stimulation without actual laser irradiation was also presented 20 times. The presentation order of the laser and sham stimuli was randomized per participant. Participants were asked to press one of the three predefined buttons on a computer keyboard to report what type of tactile sensation they perceived: no feeling, non-painful feeling, and painful feeling. In the mechanical stimulation session, each trial started with a resting period of 10 s, followed by a mechanical stimulation delivered to the right index fingertip for 0.1 s. The next trial began immediately after the stimulation. In the heat stimulation session, each trial started with a resting period of 38 s. Then an auditory instruction (a pre-recorded speech for “get ready”) was presented, and participants inserted their right index fingertip in the hold of the heat stimulation device to perceive the heat stimulus (42 °C). The temperature was then maintained at 42 ± 0.2 °C for another 4 s period. Afterward, another auditory instruction (a pre-recorded speech meaning “pull out”) was given to indicate the end of stimulation at which participants pulled their finger out of the device. The heat device started cooling with a fan to decrease the temperature to the baseline level. Participants rested their right hand during this resting period of 38 s. In the sessions for mechanical and heat stimulation, no sham stimuli were used, and self-ratings for perceived sensations were not asked.

#### Subjective evaluation

After the laser stimulation session, the participants who answered that they perceived non-painful tactile sensations from laser were also asked to verbally describe the sensations. The description was recorded and subject to a post-hoc analysis in which the subjective descriptions were classified into three categories of mechanical, electrical, and thermal sensations. The descriptions in the mechanical sensations category were further classified into five more specific categories: light touch, pressing, pricking, stinging, and spreading. If a participant’s descriptions corresponded to *n* multiple categories, the count of each of those categories was increased by 1/*n*. These counts of each category were summed over participants, and then the sum was normalized by the number of participants to obtain the frequency of the category.

#### EEG data acquisition

Scalp EEG was recorded using 16 wet electrodes with a V-Amp amplifier (Brain Products GmbH, Gilching, Germany). Electrodes were located according to the International 10-20 system (i.e., AF3/4, F3/z/4, FC1/2/5/6, C3/z/4, CP1/2, Pz, and Oz). EEG signals recorded from each electrode were sampled at 500 Hz, referenced to the right earlobe (A2) and grounded by the electrode placed on the middle of the head (Cz). The impedance of each electrode was kept below 5 kΩ during the recording session. After acquisition, EEG signals were band-pass filtered (0.5 - 40 Hz) to eliminate both high-frequency and power-line noises and then segmented into epochs of 3 s, from 0.5 s before the stimulus onset to 2.5 s after the stimulus onset. The epochs with artifact contamination were removed by setting a threshold of ±50 μV.

#### EEG data analysis

EEG channels over the sensorimotor area including C3 and C4 were selected for the spectral power analysis. For each epoch at each channel, the time-varying power spectral density (PSD) was estimated by the one-sided modified periodogram method using a 0.2 s Hamming window slid with a step of 20 ms^47^. The spectral band of interest included the alpha band (11 Hz–13 Hz) as well as the beta band (21 Hz–28 Hz). The PSD values were transformed in a logarithmic scale and averaged across each of the two frequency bands. Statistical significances of increases or decreases in the PSD values after stimulation were assessed using a *t*-test. The null hypothesis was that the mean PSD value in each time window after the stimulus onset was equal to the mean PSD value in the baseline segment, defined as a 0.5 s period before the stimulus onset. To evaluate the similarity between mechanical pressure and innocuous laser stimulations in comparison to others, pattern analysis was performed on the temporal power change of the alpha and beta oscillations over the whole brain. ERD/ERS patterns were acquired during the time interval between 0.5 s before and 4 s after the stimulus onset for all types of stimulations, except for the heat stimulation, where they were acquired in the time window of 4 s to 8 s after the stimulus onset due to the rising time. The linear-correlation coefficient (LCC) in each channel was calculated for all the possible pairs of ERD/ERS patterns from the five types of stimulations (mechanical, heat, laser with non-nociceptive, nociceptive and no feelings). Then, after determining the average LCC across the channels, we used its reciprocal to define the degree of similarity between two stimulations. By building a relational matrix from these measures and using multidimensional scaling (MDS), we obtained the 2-D spatial representation of the ERD/ERS patterns for the five types of simulations^26^. When the ERD/ERS patterns of two stimulations are more similar, the corresponding points to those two patterns are closer in this 2-D space.

For the ERP analysis, the vertex EEG channel at Cz was selected. The EEG signal in each epoch was further band-pass filtered in 0.5–15 Hz. For each subject, EEG waveforms in each case when subjects reported a noxious feeling or when they reported an innocuous feeling in response to laser stimulations were collected and averaged across epochs to generate ERPs for each stimulus. Baseline correction using a baseline period of 0.5 s before stimulus onset was carried out for each epoch data. In each ERP, the relevant negative peaks were searched within a time interval from 0.1 s to 0.3 s after stimulus onset because the negative peaks specific to nociceptive laser or non-nociceptive tactile stimuli have been reported to occur at 0.22 s for LEP (N220) or 0.14 s for SEP (N140)^31^. Then, the latency of the ERP was determined by measuring the time instant when the largest negative peak was observed within this 0.2-s period. For comparison, we also analyzed the latency of the of the ERPs in response to mechanical stimulations in the same way as above, except that we used all the EEG epochs without grouping them according to the detailed responses of subjects to mechanical stimuli. The ANOVA analysis was performed to examine the differences between the latencies of the ERPs elicited by nociceptive laser, non-nociceptive laser, and mechanical stimuli, followed by post-hoc *t*-tests.

## Additional Information

**How to cite this article**: Jun, J.-H. *et al.* Laser-induced thermoelastic effects can evoke tactile sensations. *Sci. Rep.* **5**, 11016; doi: 10.1038/srep11016 (2015).

## Supplementary Material

Supplementary Information

## Figures and Tables

**Table 1:**
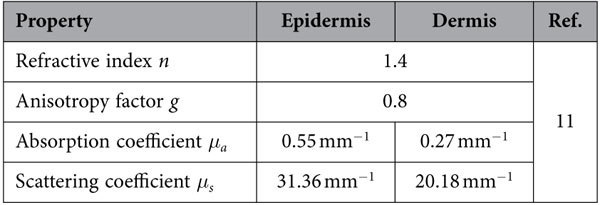
Optical properties of the human skin at 532 nm wavelength used in the simulation.

**Table 2:**
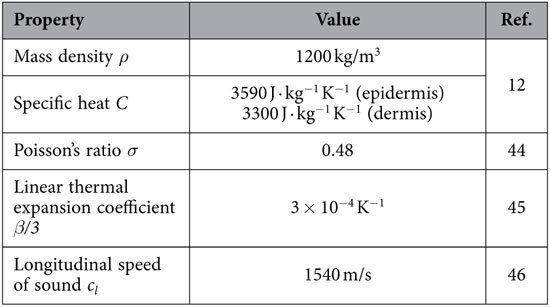
Physical properties of human skin used in this study.
