# Supplementary material for: Laser-induced thermoelastic effects can evoke tactile sensations
Source: Sci Rep. 2015 Jun 5;5:11016. doi: 10.1038/srep11016 (PMC4603782; doi:10.1038/srep11016)
Supplement: Supplementary Information [file srep11016-s1.pdf]

Laser-induced thermoelastic effects can evoke tactile sensations

Jae-Hoon Jun[1]*, Jong-Rak Park[2]*, Sung-Phil Kim[3]*, Young Min Bae[4]*, Jang-Yeon Park[5,6]*, Hyung-Sik Kim[1]*, Seungmoon Choi[7], Sung Jun Jung[8], Seung Hwa Park[9], Dong-Il Yeom[10], Gu-In Jung[1], Ji-Sun Kim[1] & Soon-Cheol Chung[1†]

[1]Department of Biomedical Engineering, BK21+ Research Institute of Biomedical Engineering, College of Biomedical & Health Science, Konkuk University, Chungju, Chungbuk 380-701, South Korea

[2]Department of Photonic Engineering, Chosun University, Gwangju 501-759, South Korea

[3]Department of Human and Systems Engineering, Ulsan National Institute of Science and Technology, Ulsan 689-798, South Korea

[4]Department of Physiology, KU Open Innovation Center, Research Institute of Medical Science, Konkuk University School of Medicine, Chungju, Chungbuk 380-701, South Korea

[5]Center for Neuroscience Imaging Research (CNIR), Institute for Basic Science (IBS), Suwon, Gyeonggi 440-746, South Korea

[6]Department of Biomedical Engineering, Sungkyunkwan University, Suwon, Gyeonggi 440-746, South Korea

[7]Department of Computer Science and Engineering, Pohang University of Science and Technology, Pohang, Gyeongbuk 790-784, South Korea

[8]Department of Physiology, Medical School, Hanyang University, Seoul 133-791, South Korea

[9]Department of Anatomy, Konkuk University School of Medicine, Chungju, Chungbuk 380-701, South Korea

[10]Department of Physics and Energy Systems Research, Ajou University, Suwon, Gyeonggi 443-749, South Korea

*These authors contributed equally to this work.

†Correspondence to Soon-Cheol Chung (e-mail to scchung@kku.ac.kr)

CON

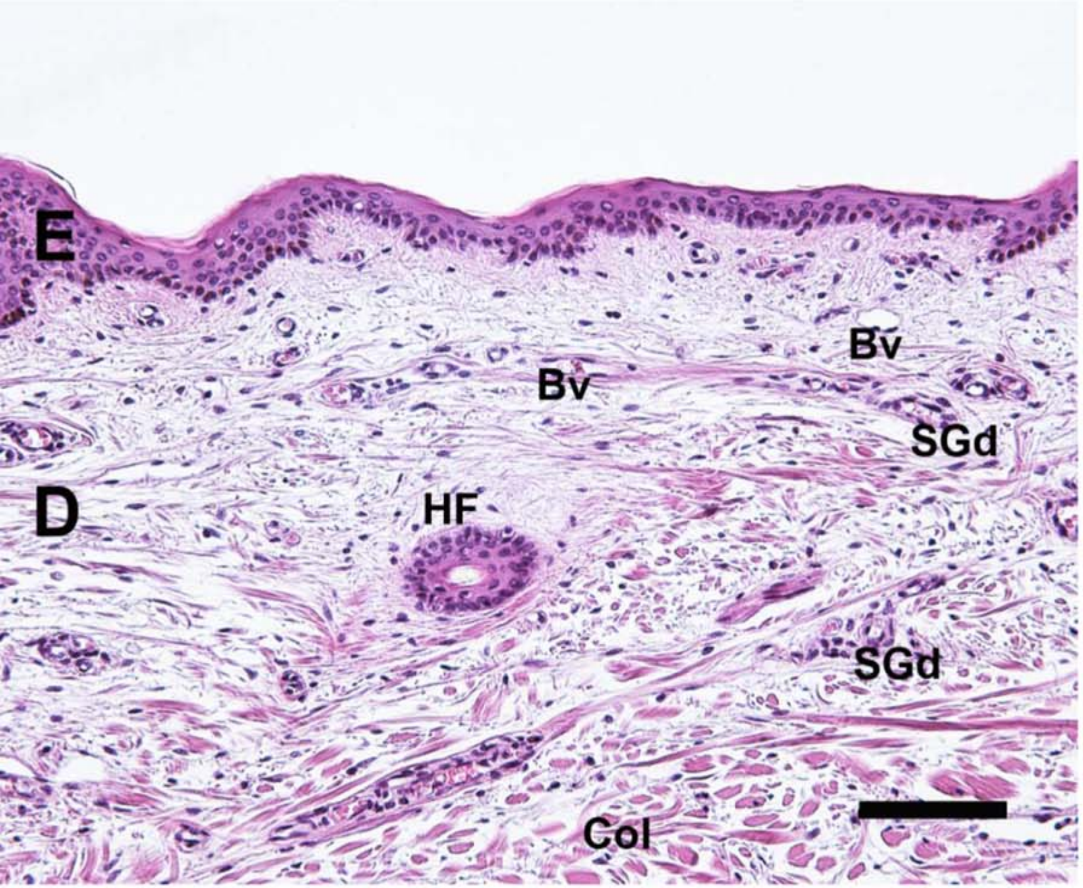

E : epidermis , D : dermis

HF : hair follicle, Bv : blood vessel

SGd : ducts of sweat gland

Col : bundles of collagen fibers

Scale bar : 100 ㎛

Laser irradiation

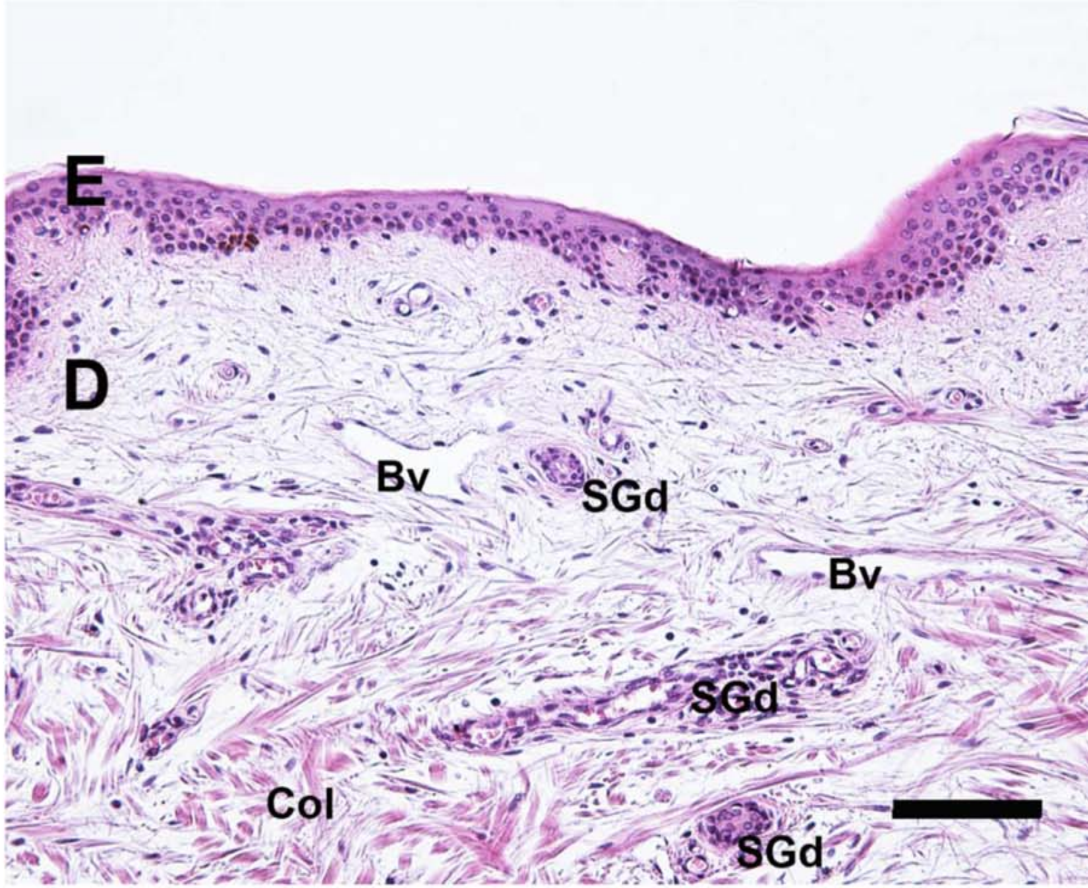

E : epidermis , D : dermis

Bv : blood vessel

SGd : ducts of sweat gland

Col : bundles of collagen fibers

Scale bar : 100 ㎛

Supplementary Figure 1. Examples of histological sections of control (left) and laser-irradiated (right) human cadaver skins. The results indicate that the laser-irradiation did not damage the skin. For histological examination, control and the laser-irradiated skins were treated in routine procedures of the histological examination and were embedded in paraffins. Paraffin-embedded specimens were sectioned at a thickness of 5 μm and were stained by the hematoxylin and eosin (H & E) staining method. All of the stained sections were photographed using a 2048x1536-pixel digital CCD camera (DP70, Olympus, Tokyo, Japan).
